# Effect of Previous Alkylating Agent Exposure on Follicle Numbers in Cryopreserved Prepubertal and Young Adult Ovarian Tissue after Long-Term Xenografting

**DOI:** 10.3390/cancers14020399

**Published:** 2022-01-13

**Authors:** Mirja Nurmio, Babak Asadi-Azarbaijani, Mi Hou, Ronja Kiviö, Jorma Toppari, Helena Tinkanen, Tiina Laine, Irma C. Oskam, Kirsi Jahnukainen

**Affiliations:** 1 Research Centre for Integrative Physiology and Pharmacology, Institute of Biomedicine, University of Turku, 20520 Turku, Finland; mihanu@utu.fi (M.N.); ronja.kivio@nina.no (R.K.); jortop@utu.fi (J.T.); 2 Centre for Population Health Research, University of Turku and Turku University Hospital, 20520 Turku, Finland; 3 Faculty of Health Studies, VID Specialized University, 0319 Oslo, Norway; babak.asadi@hotmail.com; 4 NORDFERTIL Research Lab Stockholm, Department of Women’s and Children’s Health, Karolinska Institute and University Hospital, 17164 Stockholm, Sweden; mi.hou@outlook.com; 5 Department of Pediatrics, Turku University Hospital, 20520 Turku, Finland; 6 Department of Obstetrics and Gynecology, Outpatient Department of Infertility Treatment and Gynecological Endocrinology, Tampere University Hospital, 33520 Tampere, Finland; htinkanen@gmail.com; 7 Pediatric Research Center, New Children’s Hospital, 00029 Helsinki, Finland; tiina.laine@hus.fi; 8 The Livestock Production Research Centre, Norwegian University of Life Sciences, 1432 Ås, Norway; irma.oskam@nmbu.no; 9 Department of Pediatrics, University of Helsinki and Helsinki University Hospital, 00014 Helsinki, Finland

**Keywords:** fertility preservation, children and young adults, alkylating agents, xenografting, ovarian follicles

## Abstract

**Simple Summary:**

Cryopreservation of ovarian tissue is a promising technique for fertility preservation in cancer patients at increased risk for subfertility. The International Guideline Harmonization Group recommends ovarian tissue cryopreservation for children and young adults before therapy with cumulative doses of alkylating agent at or above 6000–8000 mg/m^2^. A therapy that poses a high risk of subfertility is rarely the first-line therapy and many of the patients have already undergone several regimens of chemotherapy. The aim of our study was to assess the effects of chemotherapy exposures on the quality of cryopreserved ovarian tissue. We confirmed the harmful effects of alkylating agents on xenografted ovarian tissue and suggest that cumulative doses which are not regarded as an indication for fertility preservation in children and young adult may decrease the quality of cryopreserved follicles.

**Abstract:**

Purpose and methods: To elucidate whether previous cancer treatment affects graft recovery and follicle numbers, morphology, and development in grafts, cryopreserved ovarian biopsies obtained from 18 cancer patients aged 1–24 years with and without exposure to chemotherapy were xenografted as 1 mm^3^ fragments to immunodeficient mice for 22 weeks with exogenous stimulation. Results: Graft recovery showed no association with chemotherapy exposure, pubertal stage, or leukemia contamination. Total follicle number per recovered graft varied between 0 and 1031 in the chemotherapy-exposed and between 0 and 502 in the non-chemotherapy-exposed group. Atretic follicles formed the largest proportion of the follicle pool in chemotherapy-exposed grafts. Increased atresia correlated with exposure to alkylating agents (mean ± SD 8866.2 ± 9316.3 mg/m^2^) but not with anthracyclines, pubertal stage, or leukemia contamination. Conclusion: The observation confirms the harmful effects of alkylating agents on ovarian tissue. Therapy at the median cumulative dose of 8866 mg/m^2^ leads to the decreased quality of cryopreserved ovarian follicles in children and young adults.

## 1. Introduction

The survival of children affected by cancer has greatly improved [1]. This increased survival has necessitated increasing awareness of the long-term sequelae of the treatments on the health and well-being of cancer survivors. Aggressive chemotherapy and radiotherapy can damage the ovary and cause subfertility. A promising technique for fertility preservation in cancer survivors is cryopreservation of ovarian tissue. Post-pubertal cryopreserved tissue grafted back to the patient is able to restore ovarian function and fertility, and more than 200 live births have been reported worldwide following this procedure [2]. Furthermore, cryopreserved ovarian tissue from pediatric patients has been successfully used for the induction of puberty [3,4]. Recently, two reports have been published describing successful pregnancies stemming from regrafted ovarian tissue collected when the patients were 13 [5] and 9 years old [6].

The collection of ovarian tissue is an invasive procedure and should only be offered when there is a high risk of subfertility [7]. The International Guideline Harmonization Group recommends ovarian tissue cryopreservation for children and young adults who will be treated with cumulative doses of alkylating agent at or above 6000–8000 mg/m^2^, ovarian radiotherapy, and hematopoietic stem cell transplantation [8].

A therapy that poses a high risk of subfertility is rarely the first-line therapy in pediatric cancers, and fertility preservation may not be indicated at the time of diagnosis of malignancy. However, more girls become eligible when there is a poor response to therapy or a disease relapse. Consequently, many of the patients who have an indication for fertility preservation have already undergone several regimens of chemotherapy, including alkylating agents, prior to ovarian cryopreservation [9,10].

To date, there are only sparse data regarding the effects of chemotherapy on the development and survival of prepubertal or adolescent ovarian follicles after ovarian tissue transplantation. One study evaluated follicle density in prepubertal ovarian tissue after xenotransplantation, reporting one case with exposure to chemotherapy [11]. A recent large cohort study summarized the experience of transplanting ovarian tissue from 271 adult females with data on chemotherapy exposures [12]. Exposure to chemotherapy before cryopreservation (18.5% of all cases) was not shown to alter the results of ovarian tissue transplantation and was no longer concluded as a contraindication to tissue cryopreservation in adults.

The present study was designed to evaluate the effects of cumulative exposure to alkylating agents and anthracyclines on the survival and function of cryopreserved ovarian tissue from patients with childhood, adolescent, and young adult cancer after transplantation to immunodeficient mice. The effects of ovarian leukemic contamination and pubertal stage on graft function were also analyzed.

## 2. Materials and Methods

### 2.1. Collection of Ovarian Tissue

The study participants were recruited from the Children’s Hospital, University Hospital of Helsinki, Finland, the Department of Gynecology, Oslo University Hospital, Norway, and the Department of Gynecology, University Hospital of Tampere, Finland, as described earlier [13,14]. Adult patients from Tampere and Oslo were offered ovarian tissue cryopreservation as part of a fertility preservation program. Pediatric patients were included from Children’s Hospital in Helsinki where they participated in a fertility preservation research project. The inclusion criteria were very high risk of POI (>80%) due to the planned treatments (allogenic/autologous HSCT or radiotherapy with ovary in the field) based on the available evidence from the literature. The exclusion criteria were a high bleeding and/or infection risk. Previous exposure to chemotherapy was not a contraindication. The hospitals’ medical records were used to collect information about the patients, i.e., age, diagnosis, type of treatment, and the cumulative doses of chemotherapeutic agents. Cyclophosphamide-equivalent doses (CED) were calculated as described before [15] and cumulative anthracyclines as doxorubicin isotoxic equivalents (DIE) using the conversion factor of 1 for doxorubicin and 0.833 for daunorubicin.

The study material consisted of cryopreserved ovarian fragments from 18 patients aged 1–24 years. Six of the samples were collected before the initiation of chemotherapy and twelve after the initiation of chemotherapy. Except for one, all patients treated with chemotherapeutic drugs received alkylating agents, with cyclophosphamide as the major treatment element (Table 1).

Eight study samples were from leukemia patients with a leukemia-specific marker that enabled RQ-PCR analysis of minimal residual disease (MRD) in the cryopreserved ovarian tissue at the time of biopsy (Table 1), as described earlier [13,16].

### 2.2. Ovarian Tissue Freezing and Thawing

Ovarian tissue was cryopreserved by using the slow-freezing method with cryoprotectant agents, propanediol (PrOH) or ethylene glycol (EG), and thawed according to the cryopreservation reverse procedure, which included short incubations in medium containing a decreasing concentration of cryoprotectant EG or PrOH, as described previously [14]. All incubations took place at room temperature in a laminar flow hood under sterile conditions. The thawed ovarian cortical tissue was cut into pieces equal in size (1 mm^3^). One piece of ovarian cortical tissue was fixed in 4% formaldehyde for histology.

### 2.3. Xenotransplantation to SCID Mice

The protocol was approved by the Northern Stockholm Animal Ethics Committee (Project nr. 143/08). A total of 80 six-week-old female ovariectomized SCID mice (Charles River, Wilmington, MA, USA) were used. The mice were anesthetized, and one or two fragments of thawed ovarian cortical tissue were placed subcutaneously under the dorsal skin of the neck area on the left side of the dorsal midline, and one was grafted on the right side of the midline using a cancer implant G13 needle (Popper Precision Instruments, Lincoln, RI, USA). The fragments grafted into each animal were always from the same patient. The animals were kept in sterile conditions for 22 weeks and weighed weekly.

### 2.4. Gonadotropin Stimulation

Stimulation by intraperitoneal (IP) injection of gonadotropin (FSH: 1 IU) (Gonal F, Merck, Darmstadt, Germany) was performed every two days during the last 14 days before graft removal at 22 weeks. The animals were injected with 20 IU of hCG (Pregnyl, MSD, Halle, Germany) 24 h before graft removal.

### 2.5. Histologic Analysis and Follicle Classification

After fixation in 4% formaldehyde, the grafts were embedded in paraffin for histologic analysis, the entire tissue was serially sectioned (4 μm), and the sections were stained with hematoxylin and eosin. Two persons identified the number of recovered grafts with clearly vascularized ovarian structures and counted the number of follicles at each developmental stage in all sections. Interobserver concordance was monitored (>90%). To avoid double counting, each follicle was traced through neighboring sections.

Follicles were classified as primordial, growing (primary or secondary), or atretic follicles, as described earlier [14]. The follicles were defined as atretic if the nucleus of the oocyte or nucleus of more than 50% of the surrounding granulosa cells were pyknotic. The numbers of each type of follicles were counted and expressed as total number per graft. The estimation of the size of recovered grafts was not possible. The proportions of primordial, maturing, and atretic ovarian follicles of the total follicles were calculated.

### 2.6. Statistical Analysis

SSPS statistical software version 21 was used to analyze the data. The individual mean of follicle numbers, the ratio of growing/total follicles in frozen–thawed samples and in grafted tissues, and the individual mean of the percentage proportion of recovered grafts from each study patient were included in the analysis. The follicle data are presented as mean, standard deviation, and range. The Mann–Whitney U test was used to compare follicle numbers in tissue samples with and without exposure to cancer chemotherapy and for leukemia patients with and without leukemic MRD. The entire study material was included in Spearman’s rank correlation analysis to assess univariate correlations between follicle numbers, ratio of growing/total follicles, percentage proportion of recovered grafts, age, pubertal stage (prepubertal = 0, pubertal/post-pubertal = 1), CED, and DIE. The numbers of atretic follicles per tissue graft before and after xenografting and the ratio of growing follicles before xenografting in the entire study material were further entered as dependent variables and DIE, CED, and pubertal stage as independent variables in multiple-linear-regression analysis. Age and pubertal stage were significantly correlated, and only pubertal stage was included in the regression model. All tests of significance were two-tailed and *p*-values ≤ 0.05 indicated statistical significance.

## 3. Results

### 3.1. Analysis of Grafts

Out of 190 grafts that were xenografted in 80 SCID mice, 110 were recovered after 22 weeks (Table 1).

**Figure 1:**
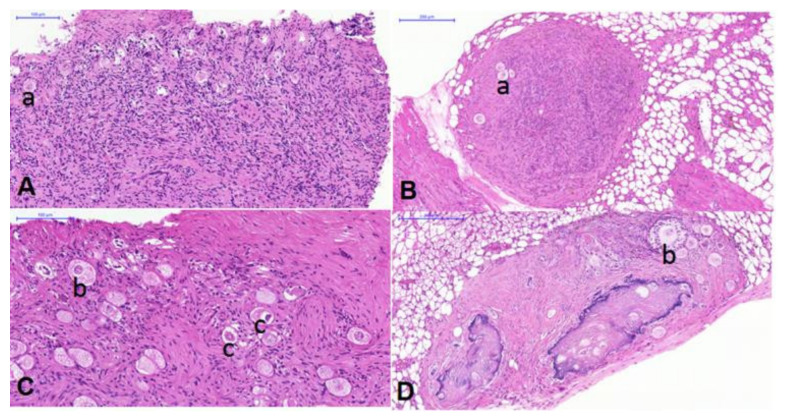
Representative images of ovarian cortex without exposure (**A**,**B**) and with exposure to chemotherapy (**C**,**D**, Case 10, CED 20840 mg/m^2^, DIE 225 mg/m^2^), before (**A**,**C**) and after (**B**,**D**) 22 weeks of xenotransplantation; (**a**) primordial; (**b**) secondary follicle; (**c**) atretic follicle.

Representative images of ovarian cortex before and after xenotransplantation are shown in Figure 1.

No difference in graft recovery was detected between samples from patients with and without exposure to chemotherapy, with and without positive leukemic MRD, or samples from prepubertal and more mature girls (Table 2). No antral follicles or masses of malignant infiltration were macroscopically observed in any of the recovered grafts.

### 3.2. Impact of Chemotherapy on Follicle Numbers

**Figure 2:**
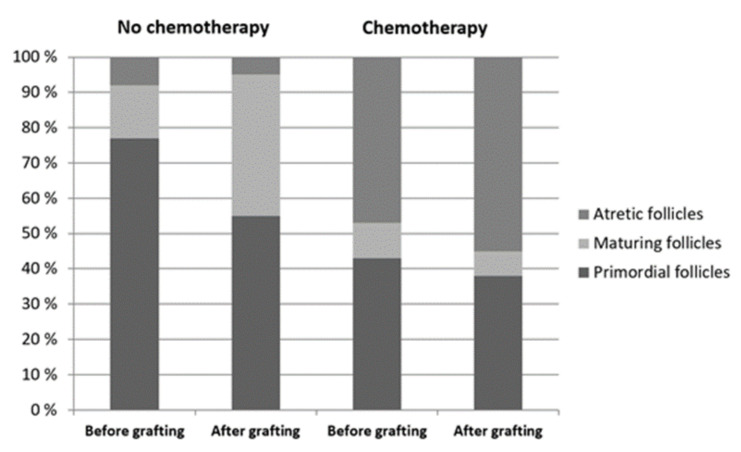
Proportions of primordial, maturing, and atretic ovarian follicles before and after 22 weeks of xenotransplantation in ovarian cortex with and without chemotherapy exposure.

Mean follicle numbers per graft before and after xenografting are shown in Table 2. Before xenografting, there were significantly more atretic follicles in samples exposed to chemotherapy than in nonexposed ovarian fragments. After 22 weeks of xenografting, no significant differences in total follicle numbers were detected between xenografts with and without exposure to chemotherapy. The ratio of growing to total follicles before or after xenografting was not affected by exposure to chemotherapy (Table 2). The proportions of primordial, maturing, and atretic follicles per graft before and after xenografting are shown in Figure 2. In chemotherapy-exposed samples, 55% of the ovarian follicles were atretic after thawing, and no increase in the proportion of growing follicles was observed after xenografting (Figure 2).

### 3.3. Impact of Leukemia Contamination on Follicle Numbers

In the samples of eight leukemia patients with a positive ovarian MRD for leukemia-specific markers, MRD did not affect the follicle numbers before or after xenografting or the ratio of growing to total follicles before or after xenografting. There were no differences in the follicle proportions between samples with positive or negative MRD.

### 3.4. Impact of Pubertal Maturation on Follicle Numbers

Before xenografting, in samples from prepubertal girls there were significantly more atretic follicles and the ratio of growing to total follicles was lower (*p* = 0.055) than in samples from more mature girls (Table 2). When samples from prepubertal girls were compared to samples from more mature girls after xenografting, total follicle count was higher, but more of the follicles were atretic (Table 2). There were no statistically significant differences in the follicle proportions between samples with positive or negative MRD (data not shown).

### 3.5. Risk Factors for Decreased Follicle Numbers

Spearman’s rank correlation analysis was performed to identify whether age, pubertal stage, exposure to cumulative cyclophosphamide-equivalent dose (CED) or doxorubicin isotoxic equivalents (DIE), or level of ovarian MRD correlated with follicle numbers in the grafts (Table 3). Before xenografting, higher age or pubertal/postpubertal stage correlated with a decreased number of atretic follicles and an increased ratio of growing/total follicles. A higher exposure to CED correlated with an increased number of total and atretic follicles and a decreased ratio of growing/total follicles, whilst a higher exposure to DIE correlated with an increased number of atretic follicles and a decreased ratio of growing/total follicles. After xenografting, the prepubertal stage correlated with an increased number of total and atretic follicles and a higher exposure to CED correlated with an increased number of atretic follicles. No correlation was detected between levels of ovarian MRD and follicle numbers.

Multivariate linear regressions were performed to adjust for differences in pubertal stage and exposure to cumulative DIE and CED. Increasing exposure to CED remained as the only independent predictor of a higher number of atretic follicles before and after xenografting (Table 4). Pubertal stage was not significantly associated with follicle numbers when adjusted for other factors in the regression model. No independent predictors were identified for the higher ratio of growing/total follicles before xenografting.

### 3.6. Leukemia Spread into the Host Animals

Altogether, 36 SCID mice were grafted with ovarian tissue from eight leukemia patients with a leukemia-specific marker. Twenty-five of the host mice received 62 grafts that were previously shown to be MRD negative [13,16] (Table 1). None of these host mice developed signs of overt leukemia. Eleven mice received 26 grafts from ovarian tissue that was previously shown to be MRD positive for the leukemia-specific marker at levels of < 0.01–0.7% [13,16] (Table 1). One of the five mice that received ovarian grafts from a patient with positive ovarian MRD at a level of 0.2% died 50 days after xenografting due to overt leukemia. Histological analysis depicted leukemic infiltration in all analyzed parenchymal organs (kidney, liver) and in bone marrow. No macroscopic infiltration was detected in brain parenchyma. Furthermore, no ovarian grafts were recovered.

## 4. Discussion

Chemotherapy with alkylating agents below the range of 6000–8000 mg/m^2^ is considered to pose a low risk for premature ovarian insufficiency, and therefore, it is not an indication for ovarian tissue cryopreservation in children and adolescents [8]. In the present study, exposure to a median dose of alkylating agents (CED 8866.2 ± 9316.3 mg/m^2^), which is at the upper limit of this low-risk-range dose, decreased the survival of follicles and increased follicle atresia after xenografting. The present study provides evidence that doses of alkylating agents that are not regarded as indications for fertility preservation in children may already harm the developmental potential of ovarian tissue. The results are in line with previous clinical reports of ovarian tissue transplantation in adults, where exposure to alkylating agents before ovarian tissue cryopreservation at a median age of 26.5 years (range 16.6–31.9) decreased pregnancy rates when compared to women treated without alkylating agents [17]. The present results from the xenografting model provide further experimental evidence that alkylating agents, in addition to causing quantitative alterations to the pool of follicles in the reserve, can lead to qualitative and functional alterations of follicles and oocytes in children and young adults [14,18,19].

Besides alkylating agents, anthracyclines are another widely used first-line chemotherapeutic agent in childhood cancers. In the present study, exposure to anthracyclines correlated with an increased number of atretic follicles and a decreased ratio of growing/total follicles in frozen–thawed samples. The observation supports our previous report about a negative correlation with the follicular and nuclear diameter of intact primordial follicles and exposure to anthracyclines [18]. After xenografting, however, only the correlation with exposure to alkylating agents remained significant. Thus, the present data provide no evidence that exposure to anthracyclines results in a further decline in the survival of grafted follicles. This observation is in line with the previous reports that exposure to nonalkylating or low-dose-alkylating chemotherapy before ovarian tissue cryopreservation does not alter the chances of success of ovarian tissue transplantation [12,17,20].

Increasing age and pubertal maturation were associated with a higher proportion of growing follicles and fewer atretic follicles in frozen–thawed samples as well as a higher number of total follicles and fewer atretic follicles after xenografting. The association between follicle numbers and pubertal maturation disappeared after adjusting for cumulative exposure to alkylating agents in the regression model. The results are in line with previous reports on higher density of maturing follicles after tissue culture with increasing age [14,21] and decreased atretic follicle counts in frozen–thawed samples [14,18].

Ovarian exposure to the alkylating agent cyclophosphamide has been suggested to trigger dormant follicle activation, resulting in burnout of the follicle reserve [22]. Our observation of a decreased proportion of growing follicles in frozen–thawed ovarian samples in girls and young women who received chemotherapy does not support the hypothesis of increased activation of dormant ovarian follicles after chemotherapy. However, it should be noted that the time since last chemotherapy prior to ovarian biopsy varied from 9 to 55 days, and it is possible that temporary follicle activation could have been missed. Instead of follicle activation, a significant depletion of all follicle types occurred during long-term xenografting. The majority of the remaining follicles were atretic and only few maturing follicles were observed, suggesting that the follicle pool in xenografts is depleted by atresia, which is further exacerbated by prior exposure to alkylating agents.

Survival of human ovarian follicles in the xenograft model is also dependent on several transplantation-related factors, such as angiogenic factors [23], hormones [24], graft site [24,25,26], and graft size [26,27]. Graft recovery in the present study showed no association with chemotherapy exposure, age, or pubertal maturation. We observed a lower graft recovery rate (50%) than previously reported (80–100%) in xenografting studies using human ovarian tissue [11,24,26]. We were not able to observe antral follicles or recover mature oocytes, which contradicts a previous report on cryopreserved adult [28,29] and prepubertal ovarian tissue [11,30] after long-term intra-abdominal xenografting and with and without exogenous stimulation. Intra-abdominal grafting has shown superior graft recovery rates in experimental studies when compared to subcutaneous grafting [24]. However, here we chose a subcutaneous location for grafting to control the location of implanted grafts and to ensure an accurate and equal graft collection at the end of the experiment. Furthermore, graft dimensions have been shown to play a significant role in reducing follicle loss at transplantation [26,27]. To standardize the grafted tissue volume between treatment groups and to analyze graft recovery and leukemia transmission in a structured manner, we used small ovarian grafts of equal size. The size of the ovarian grafts (1 × 1 × 1 mm) used was comparable to a previous study where antral follicles were successfully recovered from cryopreserved postpubertal ovarian xenografts (0.5 × 0.5 × 1 mm), but smaller than in the previously reported successful xenotransplantation of prepubertal ovarian xenografts (from 1 × 2 mm to 8 × 10 mm) [11,30]. The present observation of lack of antral and growing follicles in small grafts compared to larger xenografts [11] supports the hypothesis that fragmenting ovarian tissue into small cubes (1 mm^3^) may result in more extensive follicle loss [27]. The lack of antral follicles in xenografts could also relate to differences in patient populations. Indeed, as a major difference to previous reports, all but one prepubertal patient and four out of six pubertal or postpubertal girls in the present study were exposed to chemotherapy, compared to only one patient in the previous xenografting studies. The lack of antral follicles in xenografts from all six nonchemotherapy-exposed cancer patients is inferior compared to previously reported studies where no antral follicles were detected in xenografts of one out of four [11], zero out of one [30], zero out of nine [28], and two out of six [29] patients. The observation confirms that xenografting can potentially identify differences in the developmental potential of stored ovarian tissue.

This study provides quantitative evidence that ovarian leukemic contamination did not impair the recovery of the ovarian grafts or ovarian follicles during xenografting. This study also demonstrates that ovarian samples harboring malignant cells may have fertility potential and will be recovered as a xenograft similarly to malignant cell-free ovarian samples. Moreover, leukemia was transmitted to one out of eleven hosts that received MRD-positive, leukemia-contaminated grafts. The host with leukemia received ovarian material with leukemic MRD (0.2%) while the other four hosts that received grafts from the same patient survived, and from them, a total of six grafts without leukemia infiltration were recovered. Our finding confirms the previous reports that ovarian leukemic contamination at the MRD level carries a risk of leukemia transmission and contaminated ovaries should not be used in clinical reimplantation [16,31,32], although potential leukemic contamination did not always lead to transmission of leukemia. There are recent publications on first pregnancies in leukemia survivors after transplantation of cryopreserved MRD negative ovarian tissue [33,34]. In line with the clinical observations, all 25 hosts in the present study that received MRD negative grafts remained healthy in the 22-week follow-up. However, grafting a larger number of hosts with a larger volume of contaminated tissue or extended observation time might have increased detection of transmission.

Our study has limitations. Despite being the largest xenografted, prepubertal human-ovarian cohort to date, the studied cohort was still relatively small. The study population was heterogeneous in terms of age, and the patients were exposed to different chemotherapy agents. Ovarian fragments represented only a partial region of the ovarian cortex and might not be representative of the whole organ while the cryopreserved cortical samples were representative of ovarian tissue that is used in fertility preservation. We could only control grafted tissue volume but did not measure the exact volume of the recovered ovarian grafts. The number of recovered follicles could, therefore, only be reported per graft, not per tissue volume. It is probable that the number of host mice we used was too low, the volume of MRD positive ovarian tissue too small, and the observation period too short to detect the risk of leukemia transmission.

## 5. Conclusions

In conclusion, exposure to cancer therapy including alkylating agents at a median cumulative dose of 8866 mg/m^2^ decreased the survival of follicles and increased the number of follicle atresia in cryopreserved prepubertal and adolescent ovarian tissue xenografted for follicle maturation. The observation confirms previous clinical reports on decreased success of adult ovarian tissue transplantation after therapy with alkylating agents. In light of our findings, previous cancer therapy is not a limitation to ovarian tissue cryopreservation but delaying fertility preservation until cumulative exposure to alkylating agents of 6000–8000 mg/m^2^ will lead to decreased quality of cryopreserved ovarian follicles. Guidelines are needed to optimize the timing of fertility preservation after exposure to chemotherapy in children and young adult cancer patients.

## Figures and Tables

**Table 1:** Clinical characteristics, ovarian leukemic contamination, number of grafted and recovered grafts, and total number of follicles of each study patient. Exposure to alkylating agents is indicated by cumulative cyclophosphamide-equivalent dose (CED) and exposure to anthracyclines by doxorubicin isotoxic equivalents (DIE). Level of ovarian leukemic contamination is indicated by MRD results.

ID	Age (y)	Tanner Stage	Cryo-Protectant	Cancer Diagnosis	CED (mg/m^2^)	DIE (mg/ m^2^)	Interval from Last Chemotherapy to Ovarian Biopsy (d)	Ovarian Leukemic MRD (%)/Sensitivity of Analysis (%)	Number of Grafted/Recovered Grafts	Total Follicles Grafted/Mean per Graft *	Total Follicles Recovered/Mean per Graft
**Patients biopsied before chemotherapy**											
5	15	5	PrOH	Neuroblastoma	-	-	-	-	12/9	824	2
10	20	5	EG	Hodgkin Lymphoma	-	-	-	-	10/10	1538	207
11	23	5	EG	Hodgkin Lymphoma				-	13/4	4420	0
12	14	4	EG	Hodgkin Lymphoma	-	-	-	-	12/7	1250	57
13	22	5	PrOH	Acute Lymphocytic Leukemia	-	-	-	0.7/0.002	5/2	278	1
16	15	5	PrOH	Aplastic Anemia	-	-	-	-	12/3	2938	8
**Patients biopsied after chemotherapy**											
19	2	1	PrOH	Neuroblastoma	11,400	12,090	14	-	11/8	25,766	1031
20	1	1	PrOH	Neuroblastoma	20,840	225	55	-	11/6	7449	31
22	12	1	PrOH	Ewing Sarcoma	31,560	300	21	-	11/6	9778	514
25	20	5	PrOH	Non-Hodgkin Lymphoma	6200	300	35	-	8/5	2262	2
26	1	1	PrOH	Acute Lymphocytic Leukemia	2000	120	28	Neg/0.001	10/2	111	1
29	16	5	PrOH	Acute Lymphocytic Leukemia	2000	120	18	Neg/0.001	13/6	2396	123
30	5	1	PrOH	Acute Lymphocytic Leukemia	3600	210	11	<0.01/0.0007	9/5	2777	176
33	6	1	PrOH	Acute Myeloid Leukemia	0	300	30	Neg/0.0003	13/8	3644	177
34	15	3	PrOH	Acute Lymphocytic Leukemia	9500	260	9	Neg/0.007	13/7	2115	0
36	24	5	EG	Acute Lymphocytic Leukemia	4800	200	21	Neg/0.001	12/6	195	136
37	8	1	PrOH	Acute Lymphocytic Leukemia	6000	300	30	0,2/0.006	13/6	826	21
38	5	1	PrOH	Rhabdomyo-sarcoma	10,248	90	17	-	11/6	4983	70

PrOH, propanediol, EG, ethylene glycol, * graft size 1 mm^3^.

**Table 2:** Mean, standard deviation (SD) and range of follicle numbers per graft of cryopreserved human ovarian tissue before and after xenografting by prepubertal stage and previous chemotherapy.

	Chemotherapy			Prepubertal Stage		
	No *n* = 6 Mean ± SD (Range)	Yes *n* = 12 Mean ± SD (Range)	*p*-Value	No *n* = 8 Mean ± SD (Range)	Yes *n* = 10 Mean ± SD (Range)	*p*-Value
Age (y)	18.2 ± 4.0 (14–23)	9.6 ± 7.7 (1–24)	**0.041**	18.4 ± 3.8 (14–24)	5.0 ± 3.7 (1–12)	**0.001**
CED ^a^ (mg/m^2^)	0	8866.2 ± 9316.3 (0–31,650)	**0.001**	2248.6 ± 3416.4 (0–9496)	10,706.0 ± 10,699.4 (0–31,560)	**0.027**
DIE ^b^ (mg/m^2^)	0	212.8 ± 81.4 (90–300)	**0.001**	88.0 ± 122.3 (0–300)	208.88.7 (90–300)	**0.034**
**Number of grafts**						
Xenografted	10.7 ± 2.9 (5–13)	11.3 ± 1.7 (8–13)	0.924	11.0 ± 2.6 (5–13)	11.1 ± 1.4 (9–13)	0.573
Recovered	5.8 ± 3.3 (2–10)	5.9 ± 1.6 (2–8)	1.000	5.9 ± 2.5 (2–10)	5.9 ± 1.9 (2–8)	1.000
% recovered	55.3 ± 28.4 (25–100)	52.7 ± 12.6 (20–72)	1.000	54.4 ± 21.5 (25–100)	52.4 ± 15.1 (20–72)	0.829
**Follicle number before xenografting (per graft)**						
Total	211.2 ± 146.5 (74–464))	506.3 ± 665.4 (11–2428)	0.385	203.7 ± 135.6 (20–464)	663.2 ± 776.7 (11–2428)	0.122
Atretic	19.1 ± 38.8 (0–97)	182.4 ± 212.7 (0–717)	**0.018**	40.0 ± 54.3 (0–156)	237.9 ± 242.4 (2–717)	**0.016**
Primordial	121.2 ± 86.1 (40–280)	282.5 ± 543.6 (0–1925)	0.964	105.9 ± 88.4 (7–280)	382.4 ± 652.7 (0–1925)	0.696
Growing	20.9 ± 19.6 (8–60)	7.1 ± 7.0 (0–22)	0.083	14.9 ± 16.9 (0–60)	7.8 ± 7.9 (0–22)	0.408
Ratio growing/total (%)	10.7 ± 6.4 (3.8–20.0)	10.6 ± 22.9 (0–81.0)	0.102	9.6 ± 6.2 (0–20)	11.9 ± 28.3 (0–82)	**0.055**
**Follicle number after xenografting (per graft)**						
Total	5.1 ± 7.5 (0–19)	27.6 ± 39.8 (0–129)	0.125	6.9 ± 8.9 (0–22)	36.6 ± 46.5 (1–129)	**0.043**
Atretic	0.8 ± 1.9 (0–5)	18.7 ± 31.1 (0–84)	0.102	2.8 ± 6.6 (0–21)	25.2 ± 36.5 (0–84)	**0.051**
Primordial	4.0 ± 6.7 (0–16)	8.5 ± 15.1 (0–5)	0.385	3.9 ± 6.3 (0–16)	10.9 ± 17.8 (46–88)	0.237
Growing	0.5 ± 1.1 (0–3)	0.6 ± 1,4 (0–5)	0.892	0.8 ± 1.7 (0–5)	0.2 ± 0.4 (0–1)	0.829
Ratio growing/total (%)	33.3 ±51.6 (0–100)	8.0 ± 16.1 (0–50)	0.892	28.2 ± 41.6 (0–100)	1.8 ± 3.1 (0–9)	0.573

^a^ cumulative Cyclophosphamide-Equivalent Dose, ^b^ cumulative doxorubicin isoequivalent dose.

**Table 3:** Spearman’s rank correlation analysis of age, pubertal stage, cumulative Cyclophosphamide-Equivalent Dose (CED), cumulative anthracycline dose (DIE) and ovarian minimal-residual leukemia (MRD) and their association with follicle numbers per graft and ratio of growing/total follicle numbers and percentage proportion of recovered grafts before and after xenografting.

	Age		Pubertal Stage		CED		DIE		MRD *	
	*Rho*	*p*	*Rho*	*p*	Rho	*p*	*Rho*	*p*	*Rho*	*p*
	**Before xenografting**									
Total follicles	–0.321	0.194	–0.388	0.112	0.480	**0.044**	0.192	0.446	0.055	0.898
Atretic follicles	–0.536	**0.022**	–0.588	**0.010**	0.591	**0.010**	0.500	**0.035**	–0.343	0.406
Primordial follicles	–0.095	0.707	–0.108	0.670	0.255	0.307	–0.025	0.920	0.082	0.847
Ratio growing/total	0.473	**0.047**	0.464	0.053	–0.558	**0.016**	–0.473	**0.047**	0.164	0.699
	**After xenografting**									
Total follicles	–0.215	0.213	–0.485	**0.041**	0.311	0.208	0.247	0.323	–0.041	0.923
Atretic follicles	–0.255	0.307	–0.474	**0.047**	0.481	**0.043**	0.345	0.161	–0.291	0.485
Primordial follicles	–0.255	0.391	–0.286	0.232	0.000	1.000	0.038	0.888	–0.218	0.604
Ratio growing/total	0.047	0.853	0.172	0.496	–0.208	0.407	–0.080	0.754	–0.188	0.657
% recovered grafts	–0.111	0.661	–0.054	0.831	0.087	0.732	0.095	0.708	–0.247	0.555

Rho = Spearman’s rank correlation coefficient, * eight leukemia patients with leukemia-specific markers were included in the analysis.

**Table 4:** Multivariate-linear-regression analysis of atretic follicle numbers per graft and ratio of growing/total follicle numbers using pubertal stage, cumulative cyclophosphamide-equivalent dose (CED), and cumulative anthracycline dose (DIE) as predictors.

Outcome Variable	Predictor	B	SE_B_	*p*-Value	R^2^–adj.
**Before xenografting**					
Atretic follicles	Pubertal stage	83.232	73.397	0.276	79.7%
	DIE	–0.186	0,314	0.562	
	CED	0.016	0.005	**0.003**	
					
Ratio growing/total	Pubertal stage	12.160	11.055	0.290	39.0%
	DIE	–0.030	0.047	0.529	
	CED	–0.001	0.001	0.301	
**After xenografting**					
Atretic follicles	Pubertal stage	9.234	12,130	0.459	69.9%
	DIE	–0.041	0.052	0.437	
	CED	0.002	0.001	**0.013**	

B = Regression coefficient, SEB = Standard error.

## Data Availability

The datasets analysed during the current study are available upon reasonable request.
